# Larval migration in PERL chambers as an *in vitro *model for percutaneous infection stimulates feeding in the canine hookworm *Ancylostoma caninum*

**DOI:** 10.1186/1756-3305-4-7

**Published:** 2011-01-25

**Authors:** Daniela Franke, Christina Strube, Christian Epe, Claudia Welz, Thomas Schnieder

**Affiliations:** 1 Institute for Parasitology, University of Veterinary Medicine Hannover, Buenteweg 17, 30559 Hannover, Germany; 2 Novartis Centre de Recherche Santé Animale, St. Aubin, Switzerland

## Abstract

**Background:**

*Ancylostoma caninum *third-stage larvae are the non-feeding infective stage of this parasite and are able to infect potential hosts via different infection routes. Since percutaneous infection is one of the most important routes and skin penetration is the first step into parasitic life, an existing *in vitro *model for percutaneous migration was modified and evaluated. The main parameter used to evaluate migration was the migration ratio (migrated larvae as a percentage of total number of larvae recovered). Additionally, the skin lag was calculated, expressing the percentage of larvae remaining in the skin and therefore not being recovered. Since initiation of feeding is proposed to be an important step in the transition from free-living to parasitic *A. caninum *larvae, feeding assays were performed with *in vitro *percutaneously migrated larvae. Additionally, infective larvae of *A. caninum *were activated via serum-stimulation and feeding behaviour was analysed and compared between percutaneously migrated and serum-stimulated larvae.

**Results:**

Maximum skin migration levels of infective larvae were observed at temperatures above 32°C when larvae were placed on the epidermal side of skin for more than 12 hours. The medium beneath the skin had no effect on migration ratio, and no significant difference between the migration ratios through fresh and frozen/thawed skin was observed.

Maximum feeding levels of 93.2% were observed for percutaneously migrated larvae after 48 h incubation, whereas serum-stimulated larvae reached the maximum of 91.0% feeding larvae after 24 h.

**Conclusions:**

The PERL chamber system was optimised and standardised as an *in vitro *model for percutaneous migration. The larvae recovered after percutaneous migration showed characteristic signs of activation similar to that of serum-stimulated larvae. The observed difference in time course of resumption of feeding indicates that percutaneously migrated larvae are not identical to serum-stimulated larvae, which are currently representing the model for early parasitic stages.

## Background

Hookworms are parasitic nematodes of major importance for humans and animals. Worldwide, an estimated 740 million people are infected with the human hookworms *Necator americanus *and *Ancylostoma duodenale *[1]. Furthermore, humans can suffer from the so-called *Larva migrans cutanea*, which is a percutaneously invading larva of other hookworm species, such as the canine hookworms *Ancylostoma braziliense *and *Ancylostoma caninum *[2]. Therefore, *A. caninum *is not only important as a model organism for human hookworms, but also as a zoonotic agent. The prevalence of *A. caninum *in canids is heavily dependent on the climatic region. Currently, there are not many detailed data on the prevalence of this parasite, as most researchers do not differentiate the hookworm species due to the morphological similarity of eggs representing the diagnostic stage. Traub *et al. *analysed the faeces of dogs in temple communities in Bangkok by PCR and identified 9% of dogs with *A. caninum *single infections and 14% with mixed infection of *A. caninum *and *Ancylostoma ceylanicum *[3]. In Ethiopia, a post-mortem analysis of stray dogs detected 70% to be infected with *A. caninum *[4]. In Europe, the prevalence is much lower: in a study conducted in Denmark, 0.6% of the examined foxes harboured *A. caninum *in their intestine [5]. A similar low prevalence was determined with 0.4% for *Ancylostoma *spp. by Dubna *et al. *for dogs in the Czech Republic [6].

The infective third-stage larvae (iL3) of *A. caninum *are sheathed and represent the non-feeding free-living stage of this parasite. These larvae are able to infect potential hosts via different infection routes of which the percutaneous infection seems to be of major importance [7]. The iL3 of *A. caninum *follow the "ambushing strategy", meaning that the larvae wait for their host to come across and then actively attach to the skin [8]. To find a possible host, *A. caninum *iL3 respond to host-like stimuli, such as warmth, CO_2_, and soluble skin extracts, with directed movement [9-11]. Once attached to an appropriate host, larvae exsheath and penetrate into the host's skin. First analyses support the involvement of several proteases, such as metallo- and aspartyl-proteases as well as hyaluronidases, in these processes [12-14]. As percutaneous migration represents the first parasitic activity of the individual hookworm larva, the aim of the present study was to particularly investigate this process and its influencing factors in more detail. Therefore an existing *in vitro *model for percutaneous migration [12] was modified and Franz glass diffusion chambers [15] were adapted to the experimental needs. The usefulness of the resulting PERL chamber (**per**cutaneous **l**arval migration chamber) for investigating the process of skin penetration was validated subsequently. Since resumption of feeding is proposed to be one of the first steps during development to parasitic larvae [16], *in vitro *percutaneously migrated larvae were analysed for food uptake and compared to the serum-stimulation method for iL3 described by Hawdon and Schad [16].

## Methods

### Parasite and skin material

Infective *A. caninum *larvae (iL3) were obtained from coprocultures of faeces from experimentally infected dogs and were collected using a modified Baermann technique. Afterwards larvae were stored in H_2_O without further purification at 5 to 7°C for up to 4 weeks. Prior to skin penetration assays larvae were incubated at room temperature for 1 h to mimic normal environmental conditions for percutaneous infection. The number of larvae per ml was determined by counting at least 3 aliquots of the suspension and calculating the mean. Animal infection experiments were conducted according the animal ethics guidelines of the Lower Saxony State Office for Consumer Protection and Food Safety (approved under licence no. AZ 509c-42502-01A38). Skin from freshly euthanized beagle dogs was obtained from the University's nearby Department of Pathology.

### PERL chamber assay

**Figure 1:**
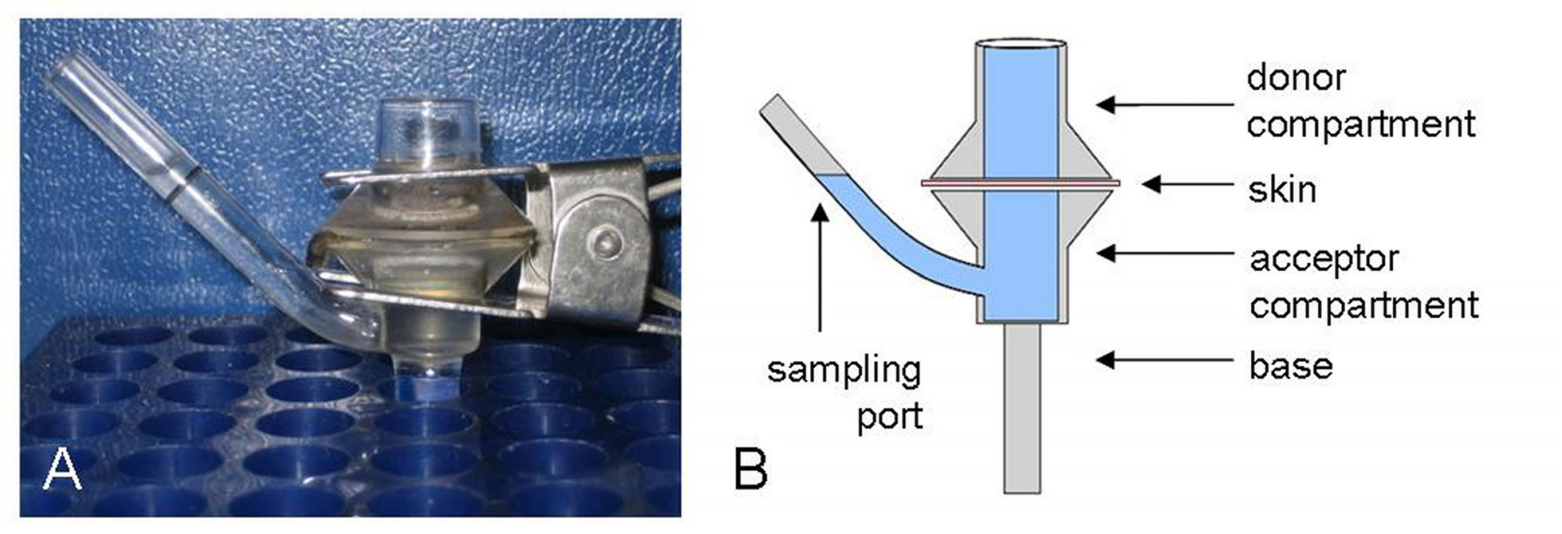
**Illustration of an assembled PERL chamber system with a schematic drawing**.

For the *in vitro *migration assays PERL chambers (Figure 1) were designed. The design was based on Franz glass diffusion chambers [15] as archetype, consisting of a donor compartment, an acceptor compartment and a clamp to fix skin between both compartments. The acceptor compartment of each PERL chamber was filled with 2 ml medium containing 50 μg/ml gentamicin, and skin was fixed between donor and acceptor compartment. Care was taken to ensure that there were no air bubbles beneath the skin. This was facilitated by the so-called sampling port of the PERL chamber. Following these preparations, the PERL chambers were preincubated for at least 30 min at the desired assay temperature in an incubator. Afterwards, 300 iL3 in 1X PBS (containing 50 μg/ml gentamicin) per chamber were added onto the skin. All migration assays were performed in darkness. After the incubation period, the liquid was collected from the donor compartment, and the compartment was washed with 0.5 ml 1X PBS. Then the chamber was disassembled, and the acceptor medium was resuspended and also collected. The number of larvae that failed to penetrate skin (i.e. larvae remaining in the donor compartment), and the number of larvae that completely migrated through the skin (i.e. larvae that were collected from the acceptor compartment), were counted separately. The main parameter to assess migration was the migration ratio, expressing migrated larvae as percentage of the total number of recovered larvae of the respective chamber:

$$\text{migration ratio}\left(\%\right)=[{\text{larvae}}_{\text{acceptor}}/({\text{larvae}}_{\text{donor}}+{\text{larvae}}_{\text{acceptor}})]\times \text{1}00$$

To take into account that larvae may start migration but are not able to completely migrate through the skin, the skin lag was determined as a second parameter. The skin lag expresses the number of larvae from not being recovered from the respective chamber as percentage of the 300 larvae added onto the skin.

$$\text{skin lag }(\%)=[\text{3}00\text{ larvae - }({\text{larvae}}_{\text{donor}}+{\text{larvae}}_{\text{acceptor}})/(\text{3}00\text{ larvae})]\times \text{1}00$$

The skin lag was used to compare parallel setups rather than setups from different experiments, as it is dependent on the actual number of larvae pipetted onto the skin, that may slightly differ between different lots of larval suspension.

Some samples of skin used during the migration assays were fixed in 10% Formalin (Roti^®^Histofix, ROTH) and finally cut and stained with Haematoxylin-Eosin (HE) to demonstrate migrating larvae.

### Evaluation of PERL chamber assay conditions

To identify optimal conditions for percutaneous migration different influencing parameters were evaluated. In each migration experiment only one of these parameters was changed. If not subjected to evaluation, the standard parameters were as follows: 37°C migration temperature, no additional CO_2_, thawed skin placed with the epidermal side facing upwards, 1X PBS as acceptor medium and 12 h incubation time. All experiments were performed in triplicate and were repeated at least once. The following parameters were evaluated:

#### Freshness of skin

Skin of freshly euthanized beagle dogs was excised and shaved since pilot experiments showed that with unshaved skin the PERL chambers were leaky. Following shaving, subcutaneous adipose and loose connective tissue was largely removed. Care was taken to avoid any injury of the skin. Prior to the assays, the pieces of skin were examined macroscopically for damages, and only intact skin was used. Skin was used either immediately as fresh skin or stored at -20°C prior to use. If frozen skin was used, skin was allowed to thaw for about 2 h at room temperature prior to preincubation.

#### Temperature

PERL chamber assays were performed at 7°C, 22°C, 32°C and 37°C.

#### Atmosphere

Assays were performed with 5% CO_2 _or without additional CO_2_.

#### Migration time

PERL chamber experiments were stopped after incubation times of 1 h, 4 h, 8 h, 12 h and 16 h.

#### Skin orientation

Skin was presented to the added larvae in the acceptor compartment with the epidermal side upwards or downwards.

#### Acceptor compartment medium

The three tested media were 1X PBS, 1X PBS containing 10% dog serum, and 0.9% NaCl solution. Serum was obtained from hookworm-naive dogs, filter-sterilized (0.45 μm) and stored at -20°C. All media were supplemented with gentamicin (50 μg/ml final concentration) to inhibit bacterial growth.

### Feeding assays

Larvae migrated through skin in PERL chambers at 37°C with or without 5% CO_2 _(percutaneously migrated larvae: pmL3 +CO_2 _and pmL3 -CO_2_), were incubated with 10% dog serum with or without 5% CO_2 _(serum-stimulated larvae: ssL3 +CO_2 _and ssL3 -CO_2_) or were incubated without serum (negative control for serum-stimulated larvae: ssL3nc +CO_2 _and ssL3nc -CO_2_). A detailed overview of the populations and their abbreviated names is given in Table 1.

**Table 1:** Overview of analysed *A. caninum *larval populations

Abbreviation	Population	Treatment
iL3	infective larvae	none; collected from coproculture
pmL3 +CO_2_	in presence of CO_2 _percutaneously migrated larvae	migration within the PERL chamber system; incubation at 37°C in presence of 5% CO_2_
		
pmL3 -CO_2_	percutaneously migrated larvae	migration within the PERL chamber system; incubation at 37°C without additional CO_2_
		
ssL3 +CO_2_	serum-stimulated larvae	incubation at 37°C in presence of 10% serum and 5% CO_2_
		
ssL3 -CO_2_	serum-stimulated larvae without CO_2_	incubation at 37°C in presence of 10% serum but without additional CO_2_
ssL3nc +CO_2_	negative control for serum-stimulated larvae	incubation at 37°C without serum but in presence of 5% CO_2_
		
ssL3nc -CO_2_	negative control	incubation at 37°C without serum and without additional CO_2_

#### Skin penetration

To produce percutaneously migrated larvae (pmL3 -CO_2_) for the feeding assays, the PERL chamber migration setups were performed with thawed skin with the epidermal side upwards and 1X PBS (containing 50 μg/ml gentamicin) as acceptor medium at 37°C without additional CO_2_. The incubation time in the PERL chambers was 24 and 48 h, respectively. Skin was removed in all assays after 24 h, for 48 h incubation time larvae remained within the acceptor medium at 37°C. In an alternative setup, the PERL chambers were incubated in the presence of 5% CO_2_. After the incubation period, larvae were recovered from the acceptor compartments and incubated with fluorescein isothiocyanat-labelled bovine serum albumine (FITC-BSA, Sigma-Aldrich), following the method of Clokey and Jacobson [17] with some modifications. Briefly, larvae were transferred to plastic tubes and an equal volume of FITC-BSA solution (5 mg/ml in 1X PBS) was added to the suspension (final concentration 2.5 mg/ml). Larvae were incubated for two hours using the initial incubation conditions and then washed three times in 2 ml 1X PBS. At least 50 living larvae per PERL chamber were analysed for FITC-BSA uptake using a Zeiss fluorescence microscope (excitation 450-490 nm, barrier 520 nm). The number of larvae that had ingested the labelled albumin was expressed as percentage of the total larvae counted. Furthermore, the percentage of exsheathed larvae was determined by counting at least 50 larvae per chamber. Each experiment was performed in duplicate or triplicate and was repeated at least once, resulting in six or more replicates.

#### Serum-stimulation

Approximately 300 *A. caninum *iL3 were transferred into one well of a 24-well deep well plate. The wells were filled up to 750 μl either with 1X PBS containing 50 μg/ml gentamicin alone (negative control) or with 1X PBS containing 50 μg/ml gentamicin supplemented with filter sterilized (0.45 μm) dog serum (final concentration 10%). Larvae were incubated at 37°C with 5% CO_2 _and without additional CO_2_, respectively, for the desired incubation period (24 h or 48 h). After incubation, an equal volume FITC-BSA solution (5 mg/ml in 1X PBS) was added to each well and the larvae were incubated for two more hours under the original incubation conditions, i.e. with or without 5% CO_2_, respectively. The number of feeding and exsheathed larvae was determined as described above. Each experiment was set up in triplicate and was repeated at least once.

All statistical calculations were performed using the SigmaStat 3.1 software package (Systat Software, Inc.). For comparison of two groups, a *t*-test was performed, followed by a Mann-Whitney Rank Sum Test in case data were not normally distributed. To compare more than two groups, One-Way ANOVA was performed and the Holm-Sidak method was used as post-hoc test. If the raw data were not normally distributed, a Kruskal-Wallis One-Way Analysis of Variance on Ranks was done, followed by the Dunn's method. A p-value < 0.05 was considered as statistically significant.

## Results

### Skin penetration studies in PERL chambers

Analysis of the impact of several experimental parameters on migration ratios resulted in the following data:

#### Freshness of skin

**Figure 2:**
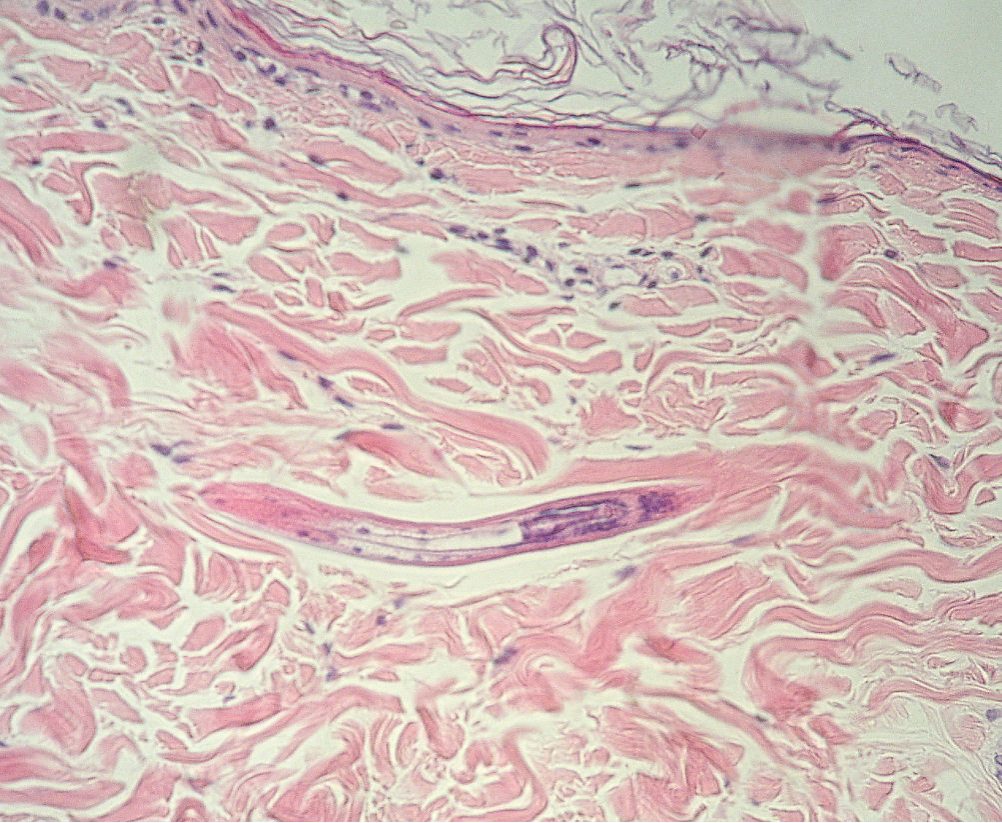
**Larvae of *A. caninum *crossing dog skin *in vitro***. Longitudinal section, HE-staining (400X magnification).

*A. caninum *larvae migrated through both, fresh and thawed canine skin (see Figure 2 for a histological section). The migration ratios of larvae did not differ significantly (t-test, p = 0.893; t = -0.138 with 10 degrees of freedom) between experiments with fresh and thawed skin that had been stored at -20°C previously. The mean migration ratios were 92.6% with a standard deviation (SD) of 5.1% for fresh skin and 93.0% (SD = 3.3%) for thawed skin. In contrast, there was a statistically significant difference (t-test, p = 0.038; t = 2.395 with 10 degrees of freedom) between the skin lags. These were 18.9% (SD = 5.3%) for fresh skin and 9.8% (SD = 7.7%) for thawed skin.

#### Temperature

**Figure 3:**
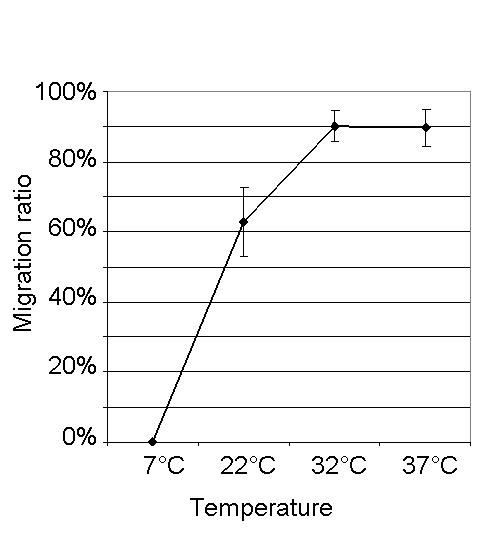
**Effect of temperature on the migration ratio of L3 after ≥ 12h of incubation**. Each point represents the mean ± SD (n=6). Larvae were incubated in PERL chambers at 7°C, 22°C, 32°C, and 37°C, respectively.

The incubation temperature had significant effects on the migration ratios (Kruskal-Wallis One-Way ANOVA, p < 0.001; H = 29.357 with 3 degrees of freedom. For illustration of results refer to Figure 3). At 7°C no larvae were recovered from the acceptor compartment. With higher temperatures the migration ratio increased: at 22°C incubation temperature, the mean migration ratio was 62.9%, and at 32°C and 37°C, respectively, the migration ratio was near 90% (detailed results including mean skin lag are listed in Table 2). The migration ratios at 32°C and 37°C were not statistically significantly different (Dunn's test, p > 0.05), in contrast to the differences to the migration ratios at lower temperatures (Dunn's test, p < 0.05). The skin lag was also significantly different between the setups (One-Way ANOVA; p < 0.001). It was highest at an incubation temperature of 22°C and significantly higher than the skin lag at 32°C and 37°C, respectively. Furthermore, the skin lag at 7°C was significantly higher than at 32°C (Holm-Sidak method, p < 0.01). The skin lag did not differ significantly between the experiments at 32°C and 37°C, neither did the skin lag at 7°C from those at 22°C and 37°C (Holm-Sidak method, p > 0.01).

#### Atmosphere

The migration ratio of larvae in presence of 5% CO_2 _was significantly lower (78.4%; SD = 13.4%; t-test, p = 0.023; t = 2.673 with 10 degrees of freedom) than the migration ratio of larvae without additional CO_2 _(94.5%; SD = 6.0%). The skin lag was not significantly different (t-test, p = 0.159; t = 1.523 with 10 degrees of freedom).

#### Incubation time

**Figure 4:**
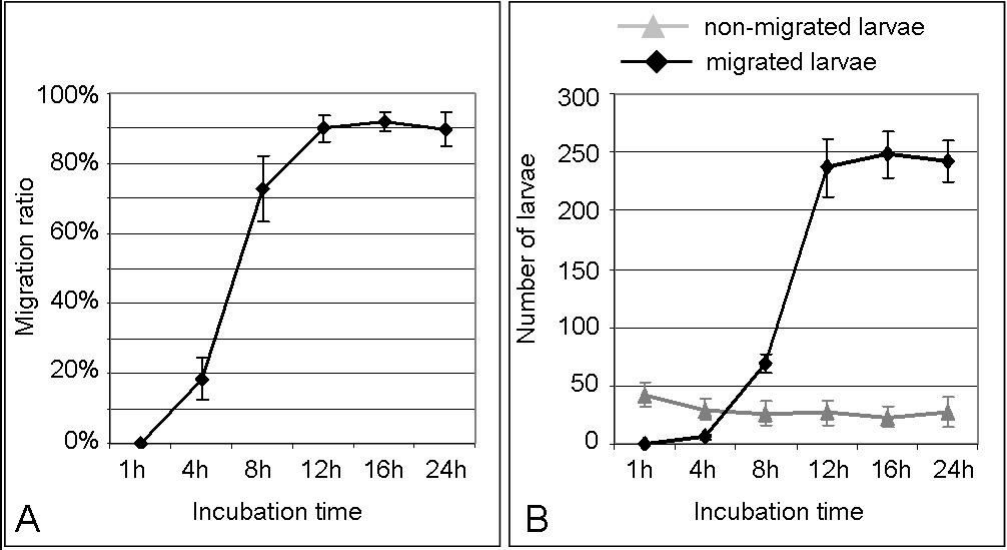
**Effect of incubation period on the migration behaviour of the L3**. Each point represents the mean ± SD (n = 6). Larvae were incubated in PERL chambers for 1 h, 4 h, 8 h, 12 h, 16 h, and 24 h, respectively. A: Migration ratio over time. B: Total number of larvae recovered from the donor compartment (non-migrated larvae) and from the acceptor compartment (migrated larvae).

The incubation time (Figure 4) during that larvae were allowed to migrate had a significant effect on the migration ratio (Kruskal-Wallis One-Way ANOVA, p < 0.001; H = 30.241 with 5 degrees of freedom) and skin lag (One-Way ANOVA, p < 0.001). After 1 hour of incubation most of the applied 300 larvae had invaded the skin, as the mean number of larvae recovered from the donor compartment was 42.33 (SD = 10.98), but the larvae could not be recovered from the acceptor compartment at that time point (Figure 4A). Therefore, the migration ratio was 0% for 1 h incubation time and increased over time (Figure 4B). The mean migration ratios were highest for incubation periods of 12 h and more (detailed results including mean skin lag are listed in Table 3). These migration ratios were not significantly different from each other (Dunn's test, p > 0.05) but from that after 1 h incubation (Dunn's test, p < 0.05). The mean skin lag also decreased from shorter to longer incubation periods. The skin lag was not statistically significant between the experiments with 1 h and 4 h incubation time and between the experiments with incubation periods of 12 h and longer (Holm-Sidak method, p > 0.01). All other comparisons detected significant differences (Holm-Sidak method, p < 0.01).

**Table 2:** Influence of temperature on migration ratio and skin lag (n = 6)

Temperature	Mean migration ratio (± standard deviation (SD))	Mean skin lag (± standard deviation (SD))
7°C	0.0% * (± .0%)	30.0% (± 18.1%)
22°C	62.9% * (± 9.9%)	39.7% ** (± 13.4%)
32°C	90.2% (± 4.4%)	11.8% (± 10.8%)
37°C	89.7% (± 5.3%)	12.0% (± 9.2%)

* Statistically significant difference to migration ratio at 37°C, p < 0.05 (Dunn's test). ** Statistically significant difference to skin lag at 37°C, p < 0.001 (Holm-Sidak test).

#### Orientation of skin

Migrating larvae preferred to migrate through skin from the epidermal to the dermal side. With the epidermal side upwards the mean migration ratio was 90.3% (SD = 4.1%) compared to 81.0% (SD = 1.7%), when larvae had to invade the dermal side instead of the epidermal side (t-test, p < 0.001; t = 5.246 with 10 degrees of freedom). The skin lag for experiments with the epidermis upwards was 15.4% (SD = 9.7%), and the lag for experiments with the dermis upwards was 28.5% (SD = 11.3%). This difference was not statistically significant (t-test, p = 0.057; t = -2.155 with 10 degrees of freedom).

#### Acceptor medium

The medium within the acceptor compartment did not have a significant effect on the migration ratio of larvae (One-Way-ANOVA, p = 0.952). The mean migration ratios were 89.5% for 1X PBS (SD = 5.6%), 89.7% for 1X PBS supplemented with 10% dog serum (SD = 4.7%), and 90.3% for 0.9% NaCl solution (SD=4.1%). The skin lag was 17.2% (SD = 9.3%) for 1X PBS, 13.2% (SD=7.3%) for 1X PBS enriched with dog serum, and 17.6% (SD = 9.4%) for 0.9% NaCl solution. There was no statistically significant difference between these results (One-Way-ANOVA, p = 0.639).

#### Experiments under standard conditions

The mean migration ratio of all experiments under standard conditions (n = 48; thawed skin, epidermal side up, 37°C incubation temperature, no additional CO_2_, ≥ 12 h incubation time, 1X PBS as acceptor medium) was 91.1%, SD = 4.6%. The mean skin lag was 15.5%, SD = 11.9%.

### Feeding assays

All tested larval populations, i.e. pmL3 -CO_2_, ssL3 +CO_2_, ssL3 -CO_2_, ssL3nc +CO_2, _and ssL3nc -CO_2 _(for an overview refer to Table 1), were vital and mobile after incubation except for larvae that had migrated through skin in presence of 5% CO_2 _(pmL3 +CO_2_). Although many larvae succeeded to migrate through the skin as described above, the majority of pmL3 +CO_2 _was dead after 48h (91.1%, SD=6.6%).

**Table 3:** Influence of incubation period on migration ratio and skin lag (n = 6)

Incubation time	Mean migration ratio (± standard deviation (SD))	Mean skin lag (± standard deviation (SD))
1 h	0.0% * (± 0.0%)	85.9% ** (± 3.7%)
		
4 h	18.3% (± 6.0%)	88.0% ** (± 3.5%)
		
8 h	72.8% (± 9.3%)	68.4% ** (± 3.5%)
		
12 h	90.0% (± 3.8%)	12.2% (± 8.9%)
16 h	92.0% (± 2.7%)	9.8% (± 9.0%)
24 h	90.0% (± 4.9%)	10.0% (± 3.8%)

* Statistically significant difference from migration ratio after ≥ 12 h, p < 0.05 (Dunn's test). ** Statistically significant difference from skin lag after ≥ 12 h, p < 0.001 (Holm-Sidak test).

#### Feeding

**Figure 5:**
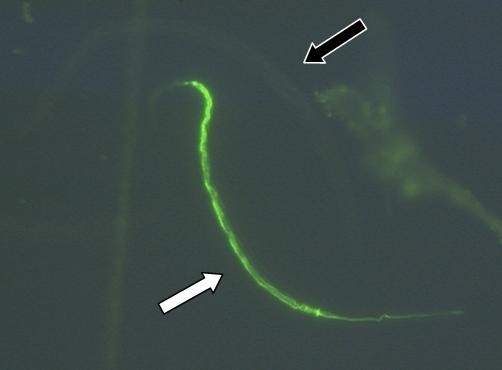
**Feeding of *A. caninum *L3 on FITC-BSA after *in vitro *percutaneous migration**. Larvae were collected after percutaneous migration from the acceptor compartment of a PERL chamber and incubated with FITC-BSA as described for the serum-stimulation protocol. No serum was added. In contrast to the non-feeding larva above (black arrow), the positive larva (white arrow) exhibits a strong fluorescence from the gut

**Figure 6:**
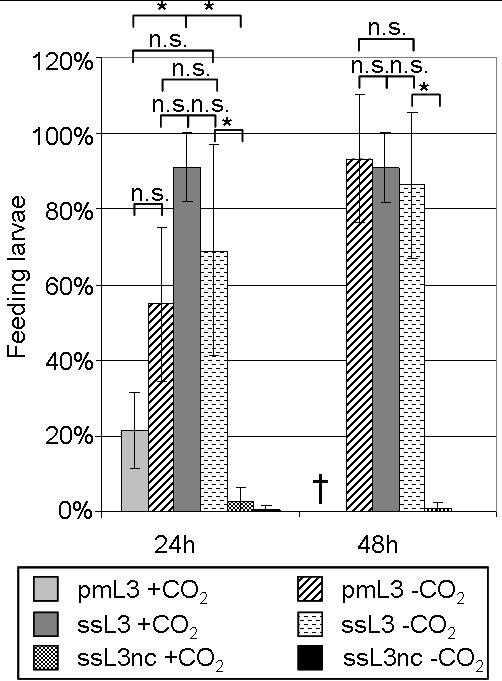
**Mean feeding ratio of the different larval populations ± SD (n ≥ 6)**. Larvae were incubated either in PERL chambers or with or without serum, and with or without CO_2 _(for detailed overview of populations refer to Table 1). The percentage of feeding larvae was determined after 24 h and 48 h, respectively, using FITC-BSA as described for the serum-stimulation protocol. For each setup, at least 50 live larvae were examined for uptake of FITC-BSA. *: statistically significant difference (p ≤ 0.05); n.s.: not significant; †: no data since most of the larvae died.

Larvae that had started pharyngeal pumping exhibited gut fluorescence as a consequence of ingested FITC-BSA (Figure 5). The differences between the populations were statistically significant (Kruskal-Wallis One-Way ANOVA, p < 0.001; H = 64.408 with 5 degrees of freedom). The population with the highest percentage of feeding after 24 h were the ssL3 +CO_2 _(usually called serum-stimulated larvae). Statistically, the ratio of feeding larvae was similar after 48 h. The feeding level of the percutaneously migrated larvae pmL3 -CO_2 _at both time points was statistically not significantly different from that of the ssL3 +CO, (Dunn's test, p > 0.05), nonetheless, the increase over time was statistically significant (Mann-Whitney Rank Sum Test, p = 0.001; T = 115.0). Detailed results are illustrated in Figure 6.

#### Exsheathment

**Figure 7:**
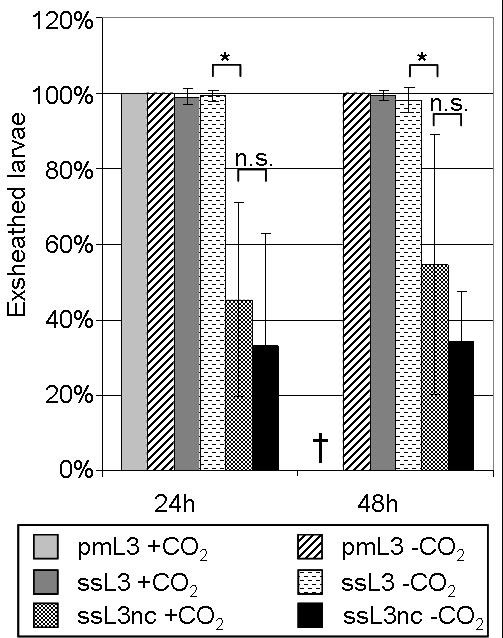
**Mean exsheathment ratio of the different larval populations ± SD (n ≥ 6)**. Larvae were incubated either in PERL chambers or with or without serum, and with or without CO_2 _(for detailed overview of populations refer to Table 1). The percentage of exsheathed larvae was determined after 24 h and 48 h, respectively. For each setup, at least 50 larvae were examined. *: statistically significant difference (p ≤ 0.05); n.s.: not significant; †: no data since most of the larvae died.

Migrated larvae (pmL3 -CO_2 _as well as pmL3 +CO_2_) were completely exsheathed, and the larvae incubated with serum (ssL3 +CO_2 _and ssL3 -CO_2_) were almost completely exsheathed. The control populations (ssL3nc +CO_2 _and ssL3nc -CO_2_) showed significantly lower percentages of exsheathment (Kruskal-Wallis One-Way ANOVA, p < 0.001; H = 69.435 with 5 degrees of freedom). The exsheathment ratios of these populations were not significantly different from each other (Dunn's test, p > 0.05). Results are shown in more detail in Figure 7. A Two-Way ANOVA with the stimulus (migration, serum, or none of them) as first factor and the atmosphere (additional CO_2 _present or absent) revealed that CO_2 _had no obvious impact on exsheathment (p = 0.487 after 24 h and p = 0.124 after 48 h). In contrast, the stimulus had a significant impact (p < 0.001 after 24 h and 48 h). Exsheathment was significantly higher upon either the serum or the migration stimulus than without an additional stimulus (Holm-Sidak method, p > 0.001). There was no significant difference between the exsheathment in migrated and serum-stimulated larvae (Holm-Sidak method, p > 0.001). No interaction was detected between the two factors (p = 0.553 after 24 h, 0.106 after 48 h).

## Discussion

### Skin penetration studies

The ability of infective hookworm larvae to enter their host has been intriguing to researchers for many years. Early experiments were performed by Goodey in 1922 [18]. Since then, migration behaviour of hookworm larvae has been subject of several studies to analyse the mechanism of skin penetration [12], to evaluate the impact of protease inhibitors and antisera [13,19-21], or as a parameter of fitness [22].

The present study aimed to establish an *in vitro *migration model as a reliable system to achieve reproducible and comparable results. Migrated larvae are often calculated as percentage of the original number of larvae, i.e. the number of larvae that had been calculated to be pipetteted. Practically, pipetting of exactly 300 larvae is not possible. To take these variations into account, in the present study the migration ratio was defined as migrated larvae as percentage of the number of larvae recovered from both compartments. This calculation excludes the possibility of migration ratios lower than 0% and higher than 100% and minimizes the effect of slightly variable absolute numbers of larvae. This is especially important to allow comparison of migration ratios from experiments using different lots of larvae suspensions. As this calculation omits larvae that started migration but did not succeed to completely migrate through the skin, the "skin lag" was introduced as additional parameter. It expresses the difference between the sum of larvae recovered from both compartments and the theoretically used total number of 300 larvae as percentage of 300 larvae. A higher skin lag compared to the respective control assay under standard conditions within the same experiment indicates that larvae for example remained in the skin and therefore could not be counted. The skin lag should be used to compare the results of parallel setups rather than from experiments using different lots of larvae suspensions.

Furthermore, the models as well as the experimental conditions vary between the different research groups which may lead to non-comparable results as these parameters might influence the migration. Therefore, the possible impact of different factors on the results of *in vitro *migration experiments was evaluated.

The use of thawed skin instead of fresh skin has the advantage of a higher practicability and flexibility. Furthermore, freezing allows using skin of one individual more efficiently, for example if there is a limit in the number of available PERL chambers, space in the incubator or simply time. The use of frozen skin also allows to obtain skin opportunistically from clinics or pathology, and to store it, so that for animal welfare reasons no purpose-collected skin from animals was needed. Consequently, comparison experiments started with this parameter. From pharmacological trials analysing transdermal absorption, different results regarding the comparability of fresh and thawed skin are known. According to Franz [15] and Harrison *et al. *[23], freezing has no effect on skin permeability. In contrast to that Ahlstrom *et al. *[24] observed differences in permeability for hydrocortisone between fresh and thawed canine skin, with a higher permeability in thawed skin. Nevertheless these authors also recommend the use of thawed skin, since even if it is a little more permissive than fresh skin, it still serves as limiting factor for diffusion and penetration of pharmaceutics. Although freezing might influence the chemical and physical status of the skin as a barrier, it could be assumed that the migration of hookworm larvae might not be affected to the same extent as the permeability for chemicals. The migration ratios in the present study were nearly identical for the use of fresh and thawed skin, and consequently no statistically significant difference was detected. These results are consistent with results presented by Matthews [25], who also observed no difference in the migratory behaviour of infective larvae of *A. tubaeforme *when using fresh or thawed skin. However, in contrast to migration ratios the skin lag in the present experiments was significantly lower when thawed skin was used. This indicates that some larvae remain in the fresh skin, e.g. after being trapped by still active immune cells, or hindered by structures, which might be destroyed by freezing and thawing. Thus, thawed skin might be a more artificial system than fresh skin. However, using the migration ratio as calculation basis for PERL chamber migration experiments, results are comparable. Because of practicability, flexibility as well as animal welfare aspects, thawed skin was used as standard parameter for all further experiments.

Another suggested important parameter concerning larval movement or skin penetration was the incubation temperature. The temperature-depending activity of hookworm larvae and other larvae is a commonly known phenomenon [9,26,27]. In the present study larvae also migrated in higher numbers through canine skin, when the temperature was closer to the temperature of the mammalian host. The highest skin lag in the present experiments was observed at 22°C. This observation is most likely due to the fact, that larvae had obviously started migration but apparently were not able to completely pass through the skin, at least not in the given time of 12 h. Most of the larvae incubated at 7°C had not started migration at all after 12 h, so the majority of them could be recovered from the donor compartment, which resulted in a lower skin lag than in the experiment at 22°C. For the experiments at 32°C and 37°C, most of the larvae were recovered from the acceptor chamber, explaining the low skin lags in these experiments. Although there was no statistically significant difference between the migration and skin lag at 32°C and 37°C, an incubation temperature of 37°C was chosen as a standard parameter since this temperature is comparable with the natural host's temperature.

Furthermore, standard PERL chamber experiments were conducted without additional CO_2 _because the presence of 5% CO_2 _led to a decreased migration ratio. Maybe this is due to the fact that under natural conditions the environment at the start of migration is ambient air containing 0.039% CO_2 _only. Interestingly, a high percentage of percutaneously migrated larvae used in subsequent feeding assays died within 48 h in the presence of additional CO_2 _whereas the other larval populations remained unaffected.

Concerning incubation time, the larvae obviously invaded the skin very quickly, but it took several hours before the majority of larvae were detected within the acceptor chamber. After 12 h incubation, migration ratios reached a maximum. Longer incubation periods did not result in higher migration or lower skin lag. From *in vivo *trials with beagles it is known that larvae of *A. caninum *need more time for skin penetration and migration than larvae of *A. braziliense *[28]. Williamson *et al. *[13] described that in their *in vitro *assays up to 100% of the *A. caninum *larvae had penetrated canine skin within 30 min and therefore could not be recovered from the skin surface. Such high a penetration was not achieved in the present experiments, and also not after pre-incubation at 37°C as performed by Williamson *et al *[13], which led to even higher numbers of remaining larvae (data not shown).. Thus, the larvae might have been activated before they were actually placed onto the skin. Kopp *et al. *[22] reported that in their experiments even 84.1% of the larvae successfully traversed canine skin within 2 h in contrast to up to 12 hours in the present setup. This rapid penetration and migration can be explained by the use of abdominal skin of very young puppies, generally about 6 weeks of age, and thoroughly removed subcutaneous tissue (S. Kopp, personal communication). Therefore, the barrier the larvae had to penetrate was very thin, whereas in the present study the skin was from dogs, which were at least several months old, and only loose subcutaneous tissue was removed. Another possible explanation could be the used *A. caninum *isolates, but three different isolates tested in the present study behaved comparably (data not shown). However, the present study revealed with approximately 86% penetrated *A. caninum *larvae within 1 h a quick invasion into the skin.

The orientation of the skin also influences larval migration and statistically significant differences were observed by testing this parameter. With the epidermal side upwards, 90.3% of the recovered larvae had completely migrated through the skin, in contrast to 81.0% when the dermal side was presented. The higher migration ratio with the epidermal side on top is not surprising since it mimics the natural conditions and larvae are attracted by the hair follicle system during migration [28]. The result that the tested acceptor media do not influence migration was expected since larvae most probably do not recognize which medium is beneath the skin, which functions as diffusion and penetration barrier [15].

Exsheathment and initiation of feeding of the third-stage larvae are assumed to be first steps in the development to parasitic stages [16,29,30]. The serum-stimulation method is commonly accepted as a model for the start of parasitic development [31]. However, Hawdon *et al. *[30] showed that feeding is not necessarily needed for development of hookworms, especially not after oral infection. Unfortunately, there are currently no data available on the percentage of feeding of *A. caninum *larvae and the time-course of the putative resumption of feeding after percutaneous infection *in vivo*. Regarding the purpose of feeding, Hawdon *et al. *[30] hypothesise that feeding is necessary after skin invasion, since the larvae have to cross different tissues (skin, lung etc.) on their way to the small intestine and therefore need much more time and energy than after being orally ingested. Therefore, the serum-stimulation resulting in the resumption of feeding presumably mimics percutaneous rather than oral infection *in vivo*, but lacks the migration step. In the study presented here the pmL3 -CO_2 _reached maximum feeding levels later than the ssL3 +CO_2_. This unequal time course may be caused by different stimuli or a different sequence of stimuli. But both, percutaneous migration and serum-stimulation induce exsheathment and feeding, two characteristics currently viewed as first steps of development into parasitic stages. However, since *A. caninum *larvae will usually not come directly into contact with dog serum *in vivo *before penetrating the skin, it might be assumed that the serum-stimulation omits important stimuli triggering further development. Thus, serum-stimulation alone may not reflect the whole truth. Compared to natural conditions, the PERL chamber percutaneous migration might be more adequate for the examination of molecular mechanisms and changes during the stage conversion towards parasitism. And indeed, larvae migrated through PERL chambers seem to be different from the infective and serum-stimulated larvae. So in current studies on gene transcription patterns in infective, percutaneously migrated, and serum-stimulated hookworm larvae, the obtained preliminary data show good evidence of different transcriptional regulations between these populations.

## Conclusions

The PERL chamber system was optimised and standardised for the percutaneous migration of *A. caninum *larvae. Currently, the model used for activation of *A. caninum *larvae from the free-living to the early parasitic stage is the serum-stimulation method. Interestingly, in this study differences were observed in the time course of resumption of feeding between serum-stimulated and percutaneously migrated larvae. This might indicate that the mechanisms of activation could be different between the two activation methods. In conclusion, the PERL chamber system is considered to be a suitable model to study percutaneous infection and to produce percutaneously migrated larvae for further molecular studies to extend our knowledge of the emergence of the parasitic way of live.

## Competing interests

The authors declare that they have no competing interests.

## Authors' contributions

DF and CW carried out the laboratory experiments and statistical analysis, and drafted the manuscript. CW participated in conceiving and designing the migration and feeding assays. CE, CS, and TS conceived and designed the study. All authors participated in data analysis and read and approved the final manuscript.
